# Optimizing Wavelet ECG Watermarking to Maintain Measurement Performance According to Industrial Standard

**DOI:** 10.3390/s18103401

**Published:** 2018-10-11

**Authors:** Agnieszka Świerkosz, Piotr Augustyniak

**Affiliations:** Department of Biocybernetics and Biomedical Engineering, AGH University of Science and Technology, 30 Mickiewicz Ave, 30-059 Krakow, Poland; aswierk@agh.edu.pl

**Keywords:** time-frequency steganography, ECG watermarking, wavelets, interpretation performance standard, CSE Database

## Abstract

Watermarking is currently investigated as an efficient and safe method of embedding additional patient or environment-related data into the electrocardiogram. This paper presents experimental work on the assessment of the loss of ECG (electrocardiogram signal) diagnostic quality from the industrial standard EN60601-2-25:2015 point of view. We implemented an original time-frequency watermarking technique with an adaptive beat-to-beat lead-independent data container design. We tested six wavelets, six coding bit depth values (including the automatic noise-dependent one) and two types of watermark content to find the conditions that are necessary for watermarked ECG to maintain the compliance with International Electrotechnical Commission (IEC) requirements for interpretation performance. Unlike other authors, we did not assess the differences of signal values, but errors in ECG wave delineation results. The results of a total of 7300 original and watermarked 10 s ECGs were statistically processed to reveal possible interpretation quality degradation due to watermarking. Finally we found (1) the Symlet of 11-th order as the best of the wavelets that were tested; (2) the important role of ECG wave delineation and noise tracking procedures; (3) the high influence of the watermark-to-noise similarity of amplitude and values distribution and (4) the stability of the watermarking capacity for different heart rates in atrial rhythms.

## 1. Introduction

Digital watermarking is a data processing method that is aimed at hiding auxiliary information in a data carrier according to steganography rules [1]. The watermark, being a digital equivalent of commonly used and well-known paper watermarks, is expected to be imperceptible (i.e., not modify the host signal message), undetectable by crypto-analytical methods and unreadable by an unauthorized user. Moreover, a watermarking scheme should be optimized for low computational complexity to run in real time on power-limited platforms, such as wireless sensor networks. The watermarking technique was originally proposed for images, however recently various data types have been reported as hosts for embedded data. In general, no assumptions are made about the secret (also called the watermark or embedded data) and the carrier (also known as the host or cover signal). Usage of watermarking for medical images and signals offers embedding of sensitive patient data into the record and prevents accidental confusion of the link between signal and identity. The original meaning of making the secret ‘invisible’ is also specifically interpreted here as: the medical content of the carrier should not be altered by the watermarking process.

One of such a particular host type is the electrocardiogram signal (ECG). In its digital form, it is a discrete-time sequence of quantized values that are collected at a sampling frequency of double the highest component that is present in the signal (e.g., Nyquist frequency) to satisfy sampling theorem without aliasing. However, the sequential occurrence of various bandlimited components of the ECG is more or less foreseeable thanks to physiological limitations [2], which allows for the prediction of the instantaneous bandwidth of the signal based on the detection of its particular components (also called ‘waves’). Moreover, the detection process relies on ECG segmentation that is performed by numerous algorithms that are developed for the purpose of medical diagnostics (e.g., [3,4]). The segmentation process reveals time intervals where the difference of the instantaneous bandwidth of particular ECG components and constant Nyquist frequency opens an extra space with no cardiac components, which may host supplementary data instead of intrinsic noise [5].

Digital watermarking is usually referred to in the context of data security [6,7] as it is an optimal technique to embed a secret message into the open content. Several applications have been targeted to protect confidential patient information and to provide authentication data of examination staff and equipment [8,9], patient medication or even authorization-dependent access to the quantitative results of diagnostic measurements [10,11]. A survey of such methods can be found in [12]. Two additional features of the ECG watermarking are worth highlighting here: (1) the supplementary data are persistently embedded into the ECG structure without a risk of accidental loss and (2) the supplementary data can provide a wider interpretation context from new sensors with the use of existing transmission and storage infrastructure and without challenging the backward compatibility of the ECG record.

The contribution of this paper is threefold: (1) it presents a watermarking technique based on the local variability of the ECG bandwidth; (2) it provides a systematic review of the performance of time-frequency ECG watermarking with different wavelets with the use of industrial quality reference; and (3) it proposes a simple yet effective method of adaptation of encoding bit depth to local variation of noise level. The remaining part of this paper is organized as follows: Chapter 2 provides a survey of the most important related works, Chapter 3 gives an outlook of the irreversible watermarking process, Chapter 4 presents the industrial standard for ECG measurement performance, Chapter 5 describes the experiment setup and results and Chapter 6 contains a discussion.

## 2. Related Work

A generalized review of watermarking techniques that had been applied to various biosignals was provided by N. Dey et al. [7], and the paper by J. Yoga Priya and R. Suganya [13] is specifically focused on steganography methods using the ECG as a carrier signal. We refer the interested reader to these papers, and limit the review hereafter to contributions that substantially supported our ideas.

Engin et al. [14] proposed the use of discrete wavelet transform in the watermarking technique and investigated ECG signal distortions for four frequent beat types under different noisy conditions for different wavelet functions. The percentage of undetectable beats was rather high and the signal-to-noise ratio (SNR) increased significantly in the watermarking process.

In the proposal by Kaur et al. [15], the watermark that composed of 15 digits was first embedded in low frequency chirp signal and was then coded into the ECG. Despite low capacity of such a watermark, the method is worth mentioning because it proposes a reversible watermarking. After the encryption, the ECG showed considerable distortion and is thus not diagnostable, however after the watermark extraction, a bit accurate carrier was recovered. The reversible watermarking was recently advanced and generalized to other biomedical signals in the paper by Shiu et al. [16].

Ibaida et al. proposed an efficient method of low complexity to increase the integrity of the ECG and patient meta-data. In the conference paper [17], they presented a method with a Least Significant Bit (LSB) watermarking algorithm which was then combined with encryption and scrambling techniques to protect confidential patient data [18]. The method is simple enough to be implemented in a point-of-care telemetric sensor. At the same time, it provides stegano ECG that is diagnostable for any reader, while access to personal data needs authorization. The authors also found that “changing some parts of the ECG signal will not affect the overall utility of the ECG signal”, however they did not verify this against the industrial standard for ECG interpretation quality. These ideas correspond to a general principle of steganography [19] and were also followed in our previous research [20].

Integrating the electrocardiogram watermarking and compression in a single time-frequency process was proposed by Tseng et al. [21]. Unlike in other approaches, the watermark was embedded in the lowest-frequency coefficients, while the high-frequency part of the wavelet decomposition was shrunk. The method was proven robust, however compression efficiency was only of the order of bit-accurate methods and the quality of the compressed ECG signal was significantly affected.

Recently, Jero and Ramu studied the watermarking of arrhythmic ECG signal [22] and proposed replacing regular wavelets by curvelets-based transform to hide valuable patient information into the ECG signal [23,24]. Their paper also proposed an adaptive selection of the watermarks location, however without relation to the ECG medical content. The secret information was then coded into coefficients of values that were close to zero in the high-frequency sub-band.

Wang et al. [8] improved the original approach of S. Stankovic, which was targeted to audio signals [25] and Sankari which was targeted to hosting additional point-of-care data in the ECG [26], and proposed yet another scheme that was based on the unified embedding-scrambling method. This approach guarantees the security of secret information and high quality of retrieved ECG signal. Similar approaches were also presented in the paper by Awasarmol et al. [27].

Finally, Kavya PremChandran et al. [28] and Liji et al. [9] addressed a very practical issue of the time-frequency watermarking by proposing the usage of the integer-to-integer wavelet transform to avoid truncation of the floating point values of the coefficients that may result in a loss of information using forward/inverse transformations.

## 3. Irreversible Watermarking Process 

### 3.1. The ECG Steganography Scheme 

The watermarking process starts with a limited ECG analysis process that is performed in time domain, and aims at the detection of heart beats and segmentation of P, QRS and T waves in each cycle. At this stage, it is important to notice that:

- the ECG is usually a multichannel signal (3–12 leads) with wave borders that are common to all leads,
- the ECG is considered quasi-periodic, however in practice, particularly in the presence of arrhythmia, each heartbeat has to be considered individually.

For the detection and wave segmentation task we used procedures from certified diagnostic software (Ascard 6^®^ by Aspel S.A., Poland). According to manufacturer-independent test results, the software meets the international industry standard for ECG measurement performance (see Chapter 4). For each heartbeat, the following meta-parameters were used in the watermarking process: P-onset, P-end, QRS-onset, R-peak, QRS-end and T-end.

**Figure 1:**
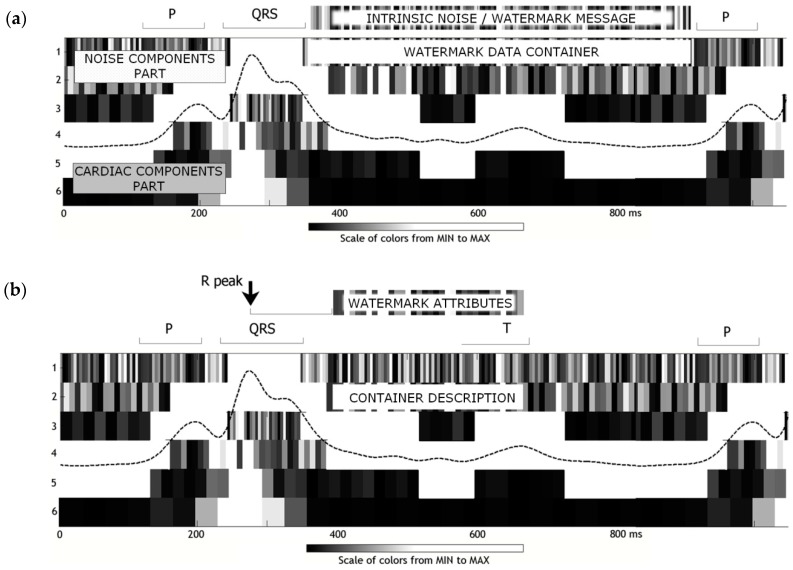
The instantaneous bandwidth of one heartbeat dividing its time-frequency representation into two parts: cardiac components and noise components with the resulting design of data containers: (**a**) the data itself are stored in the 1st scale of the bandgap with specified bit depth (e.g., 3 bits/sample); (**b**) the data description is stored in the 2nd scale of the bandgap with LSB method [17].

The essential part of the watermarking process is then made in the time-scale representation of the signal and is thus embraced by two complementary steps of forward and inverse wavelet transform of the same type and order. Its time-scale part consists of three principal stages [29]:

1. Analysis of the bandgap and creation of data containers. The maximum length of the container extends from the QRS-end to the P-onset point of the adjacent heartbeat (Figure 1a). The actual length of the container was shortened by safety margins of 60 ms (15 samples) which were added at each end to avoid interference of the watermark with high frequency components of the carrier expected in P-onset to the QRS-end section. The data container area is located in the first scale of time-scale ECG representation.
2. The watermark data stream is then transformed to *n*-bit representation (*n* ∈ {1…5}) and chopped accordingly to the length of the destination data container. The remaining part of the watermark is queued for encoding in the next available container. The watermark samples directly replace time-scale values in the container area. When watermark samples are too few to entirely fill the available container space, the length of the container is updated.
3. The container description is encoded in the second scale of time-scale ECG representation with 1-bit representation (i.e., an LSB coding [17]) in a fixed structure (Figure 1b). The description consists of 18 bits of the following meaning: the start pointer relative to precedent R-peak (6 bits), the length (9 bits) and the coding bit depth (3 bits). It begins at the point delayed by 96 ms (12 samples) from the R-peak and lasts for 144 ms, which is short enough to avoid interference with medium-high frequency components of the P wave from the next heartbeat. Such arbitrary selection guarantees the independence of the description field and the ECG content and simplifies the decoding process.

**Figure 2:**
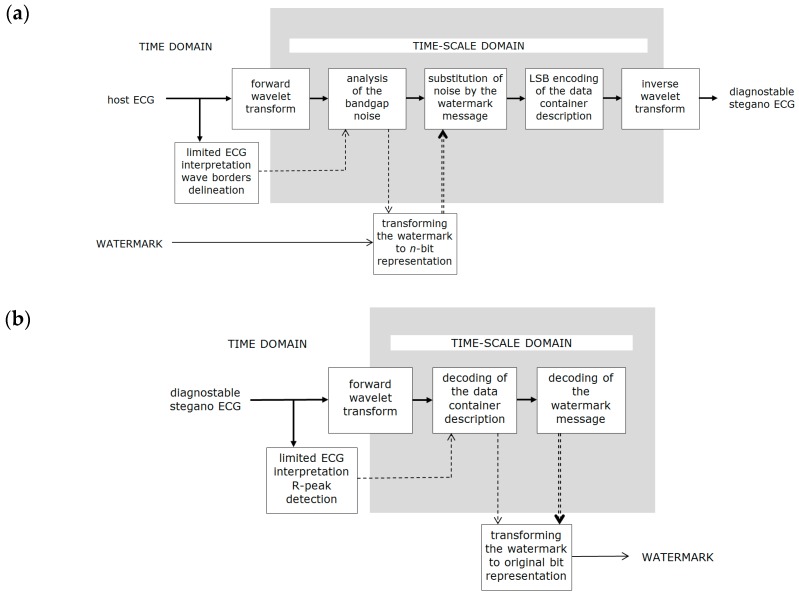
Irreversible watermarking process (**a**) watermark embedment; (**b**) watermark extraction.

The extraction of watermark also includes preliminary analysis of the ECG, however this time a R-peak detection procedure is sufficient to provide synchronization to the container description structure. The watermarked carrier preserves all diagnostic features of the ECG and, therefore, is not modified by the decoding scheme. Only the forward wavelet transform is necessary, however it has to use the same wavelet (type and order) as the watermarking scheme. Watermark embedment and extraction schemes are presented in Figure 2.

**Figure 3:**
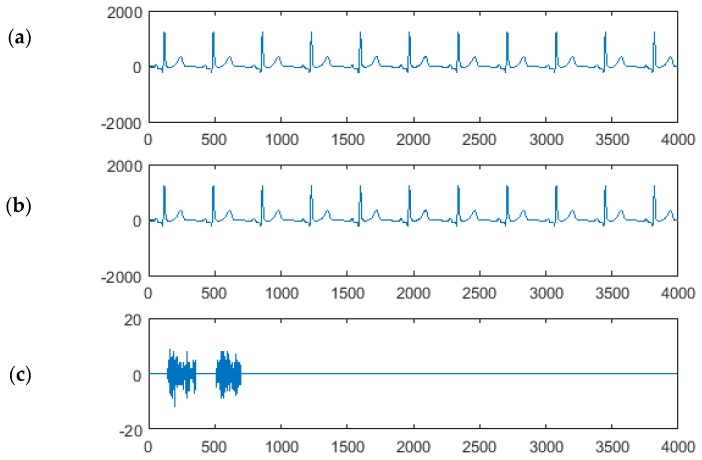
Signals in irreversible watermarking (in this example: CSE MA001, lead I, sym6 wavelet, textual watermark in 2-bit representation): (**a**) clean ECG (electrocardiogram signal); (**b**) watermarked ECG and (**c**) difference due to watermarking (the amplitude scale is 100 times lower); a 47 character watermark requires 188 2-bit samples and has been divided into two data containers of adjacent heartbeats.

The data description structure begins at a fixed distance to the detected R-peak. It provides all necessary container description (i.e., pointer to the data container, its actual length and bit per sample value). This allows for unambiguous extraction of the watermark samples, followed by concatenation of data and transcoding to the original bit representation. The extracted watermark is always bit accurate to the original, however once the ECG is watermarked, it contains the watermark permanently (irreversible watermarking) unless purposely erased (Figure 3). 

### 3.2. Adaptation of Coding Bit Depth 

Noise is omnipresent in any real-life measurement. In electrocardiography, the term of noise refers to any component of the record that is not related to cardiac activity. The principle of irreversible watermarking is that the secret was undetectable by statistical methods. The watermark should thus mimic the noise as good as possible in terms of amplitude and distribution of values. Since the intrinsic ECG noise varies from one heartbeat to another, best results are expected with the use of a noise tracking procedure that controls the adaptation of bit representation value in individual data containers. 

For this purpose, at the first stage of the watermarking process, we apply a procedure that scans the container area (i.e., first scale of time frequency representation between the QRS-end and P-onset points) and measures the noise level *v*_p-p_. This determines the coding bit depth *n* (i.e., number of bits per a watermark sample) that is used for watermark data storage in the container as equal to ⎡log_2_ (*v*_p-p_)⎤. Consequently, the value of *n* regulates the data capacity of each individual container. In practice, the length of the S-P section varies from beat to beat and the noise level also varies from channel to channel.

## 4. Industrial Standard for ECG Measurement Performance 

An international multicenter project entitled ‘Common standards for quantitative electrocardiography’ (CSE) aimed at quantitative standardization of ECG measurement procedures and ended in 1989 [30] with establishing a global reference set of electrocardiographic records (500 Hz, 0.25 µV resolution) that were accompanied by their basic measurement results. This set is known as CSE Multilead Database. Besides 125 15-lead 10 s records, it provides information of the reference beat position and all wave border positions: P-onset, P-end, QRS-onset, QRS-end and T-end calculated by 20 automatic interpretation software sets from Europe, the US, Canada, and Japan and two human experts. The Dataset DS3 contains two subsets:

– original records ‘MO’ lasting for 10 s with natural slight beat-to-beat differences, where the most representative beat is distinguished as a reference and is accompanied with measurement results,
– artificial records ‘MA’ built of the repetitions of the original reference beat as many times as necessary for a 10 s section, thus having all heartbeats identical.

Testing the wave delineation accuracy is not possible with MO files, since the results vary for individual heartbeats. The concept of reproducing the reference beat in MA files makes all beats identical and the wave delineation results independent on beat selection. Despite their misleading name, the MA files are live-recorded and are not mathematically synthesized sections of ECG. The results of each point type are sorted in time-ascending order, allowing for individual calculations of the average and standard deviation values, however not for ranking the participating programs. Testing a new software against the CSE Database consists of comparing each calculated wave border position to the 20 corresponding reference results in 125 MA files [31]. This allows us to determine the (1) average difference of wave border position from the center of the reference results; (2) deviation of this difference; (3) accuracy rank of tested software among the reference programs and to detect files of significant contribution to this error. For these results, the CSE Database proved to be a valuable tool for interpretive software engineering, fine tuning and evaluation.

In order to establish a worldwide quality standards for automated interpretation of electrocardiographic records, the International Electrical Committee published a normative document IEC 60601-2-51 [32] (currently referred to as EN60601-2-25:2015 [33]) specifying required parameters to test, testing conditions and acceptance threshold values. In the following years, this document was adopted in the majority of countries worldwide as a legal background for qualifying medical grade products to the market.

As principal diagnostic results in electrocardiography are based on time intervals, the EN60601-2-25:2015, among other parameters, specifies four intervals: P wave duration, P-Q interval, QRS complex duration and Q-T interval, of which the measurements have to comply with specified accuracy requirements. These intervals can be directly calculated from wave border points in a heartbeat. The EN60601-2-25:2015 also specifies a set of 100 biological test files which is a subset of the CSE Database with all extra-atrial rhythms excluded (i.e., with no P wave). Additionally, the normative document requires the amplitude measurement deviations to fall below ±25 µV (or 5% for amplitudes exceeding 500 µV). In the watermarking process, the amplitude accuracy can easily be controlled by adjusting the number of bits per watermark sample, which sets a compromise between amplitude accuracy and capacity of data containers. This issue was tested in one of our previous works [11] in a throughput-demanding setup which was capable of embedding the physical load data (1620 bps) of a stress test that was made at home into a 3-lead Holter ECG signal. The average error of ST-segment elevation or depression value of ±15.7 µV shows good conformance with the EN60601-2-25:2015 standard.

Since the irreversible steganography alters the content of the ECG signal, most authors make evaluation of their methods based on the Percent-Residual-Difference (PRD), which is loosely related to the possible deterioration of diagnostic value. Otherwise, using an IEC-certified interpretive software for evaluation and comparing respective results of clean and watermarked carriers, we can easily assess whether the watermarking process is suspected to cause confusion about medical findings. In the experimental part of the research that is presented beneath, we consider the medical content as fully preserved if all interval results from both clean and watermarked ECGs fall within the IEC-specified accuracy margin. To the best of our knowledge, no other research on ECG steganography used the industrial CSE/IEC-based standard as an evaluation tool. 

## 5. Experiment Setup and Results

### 5.1. The Experiment Variables and Workflow 

The proposed watermarking technique (see Chapter 3) has been implemented in Matlab (MathWorks Inc., Natick, MA, USA) and has been run in a series of experiments. For each set of watermarking parameters, three steps were performed:

1. watermarking was performed with all 100 IEC/CSE test files (MA records which had heartbeats that were all identical),
2. each clean or watermarked ECG signal was fed into the interpretive software (provided to us as an external executable file),
3. results of wave border detection were processed and checked for compliance with IEC accuracy requirements.

**Figure 4:**
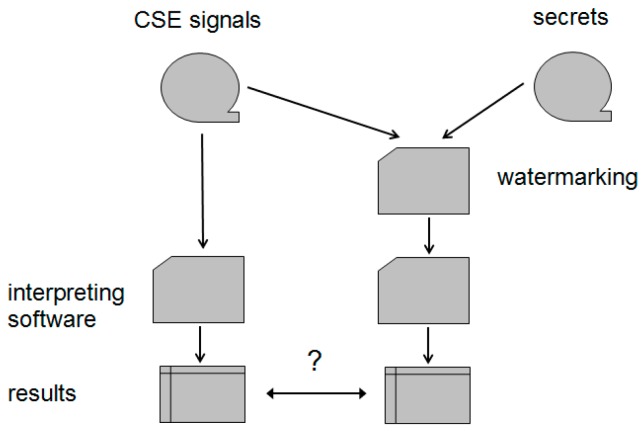
Experiment workflow.

We also implemented the watermark extraction procedure to check the accuracy of the recovered watermark, however in all cases, we got identical data streams as expected since its bit accuracy is guaranteed by the method. Figure 4 presents the experiment workflow. 

We used the following watermarking parameters (i.e., experiment variables) to iterate through:

- wavelet type and order,
- coding bit depth (number of bits per watermark sample in time-frequency representation),
- watermark content (numerical and textual),
- host ECG content.

We used three mother wavelets that were commonly applied for ECG compression due to their similarity to the QRS complex [34,35]: Daubechies (db), Symlets (sym) and Biorthogonal (bior). For each family, we applied two wavelet order (i.e., filter length) values for which the 2nd scale of time-frequency decomposition is best corresponding to the length of QRS complex. The wavelet order is also a compromise between the strength of energy separation between time-frequency scales (higher is better) and energy spread on neighbor samples in time domain (lower is better). The last issue is a reason for applying the safety margin of 60 ms at both ends of data containers. It helps to avoid interference of the watermark samples with possible high frequency components of the carrier in P-onset to QRS-end section at the price of data container capacity. In the experiment, six wavelets were iterated through: db5, db10, sym6, sym11, bior2.4 and bior4.4.

In the watermarking process, the secret message is first tailored into *n*-bit samples that replace intrinsic noise in the data container area in the time-frequency ECG representation. The form of watermark message is expected to mimic the noise as closely as possible, making the watermark undetectable and preserving the original medical content of the host ECG. Since the original CSE records are sampled at 500 Hz with an amplitude resolution of 0.25 µV per LSB, we tested the watermarking process with *n* ∈ {1…5} bits per sample, corresponding to noise peak-to-peak amplitude of 0.25–8 µV. In addition to a fixed *n* value, being conscious of noise level variability, we implemented the automatic detection procedure (see Chapter 3.2) and tested the watermarking with a beat-to-beat adaptation of watermark coding bit depth.

To make the watermarking undetectable, the watermark message should mimic the noise not only by the amplitude, however also by the distribution of sample values. We assumed the noise shows Gaussian distribution, which is not the case of ASCII text codes nor of numerical values. Therefore, it was worth testing whether the content of watermark influences the performance of the method. Two separate strings of equal length codes were used in the experiment as test watermarks: one composed of text message and the other being a sequence of digits. 

Finally, the electrocardiogram being itself a carrier of diagnostic message, offered various conditions for watermark embedment. Low heart rates enabled us to build long data containers with longer intervals between them, while high heart rates allowed for more frequent but shorter containers. Various arrhythmia types showed a difference of P-onset to QRS-end interval contribution, and in ventricular beats, the P wave was absent. For this reason, we also found it reasonable to test the dependence of the watermarking performance on the heart rate, however we left the extra-atrial beats (i.e., without P wave), which was also not included in the IEC-recommended subset of CSE files and was beyond the scope of our research. 

Table 1 summarizes the average difference values between the results on IEC interval length (P wave duration, P-Q interval, QRS complex duration and Q-T interval) which were calculated with the reference software from 100 clean and watermarked ECGs from an IEC recommended set. 

### 5.2. Results for Different Wavelets

**Figure 5:**
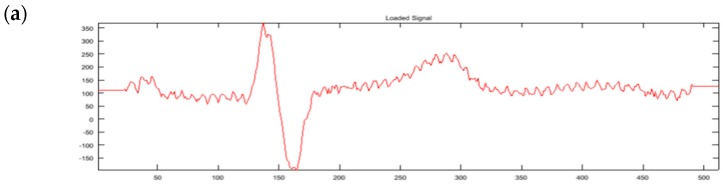

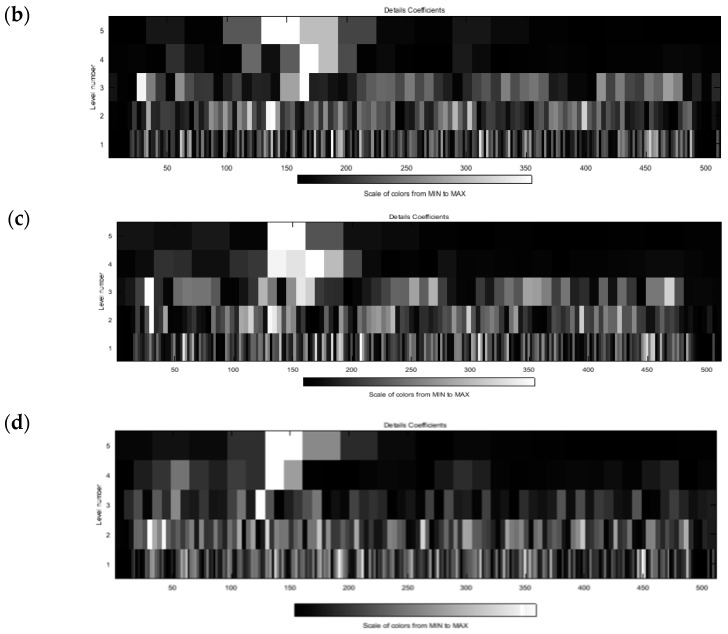
Difference of time-frequency representation with use of different wavelets (example of CSE MA001). (**a**) clean carrier; (**b**) time-frequency representation with db5 wavelet; (**c**) time-frequency representation with db10 wavelet; (**d**) difference between time-frequency representations with db5 and db10 wavelets.

By applying different wavelets, we obtained different time-frequency decomposition results. Their pairwise comparison was made separately for each CSE file (the cycle length differed between files) and required setting the *dwtmode* parameter to periodization in order to make time-scale representation size independent on wavelet filter length. An example case of difference between decompositions with db5 and db10 wavelets is presented in Figure 5, however similar results were recorded for all other cases.

Obtained results show that:

- the wavelet used has an impact on the decomposition content and
- the wavelet used for watermark embedment and for extraction must be the same.

The wavelet type-oriented analysis of Table 1 justifies the ranking of the watermarking performance presented in Table 2.

**Figure 6:**
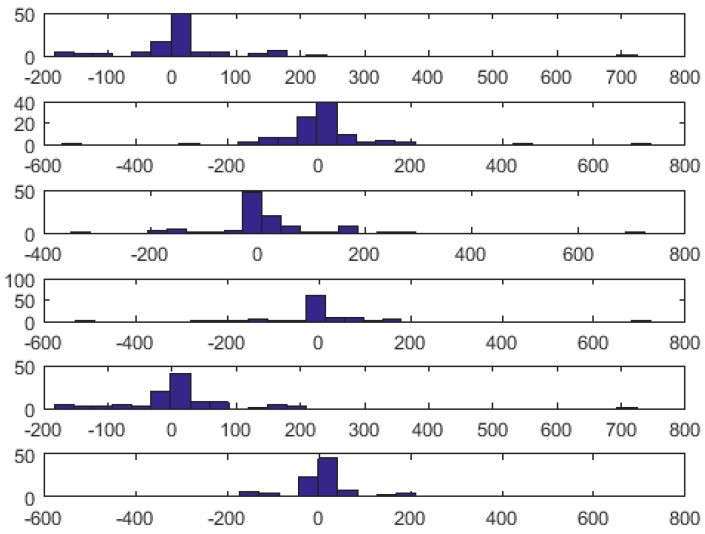
Histograms of QRS length error distribution for 1 bit depth watermark coding (worst case) with different wavelets (top to bottom): db5, db10, sym6, sym11, bior2.4 and bior 4.4.

In order to reveal possible wavelet-related differences between the interval measurement errors, for each of four interval types (i.e., P, P-Q, QRS and Q-T), we did 15 pairwise comparisons of 100-elements error sets between any two tested wavelet types using a *t*-test. In all 60 cases, we got the value of 0, meaning that no statistically significant difference exists between the error sets. An example histogram of QRS length error distribution for 1 bit depth watermark coding with different wavelets is presented in Figure 6.

### 5.3. Results for Different Coding Bit Depth 

As Table 1 shows, bit depth that is used for watermark coding plays a crucial role in the possible deterioration of ECG content. The summary of interval measurement errors that were obtained for particular values of bit depth coding is presented in Table 3.

**Figure 7:**
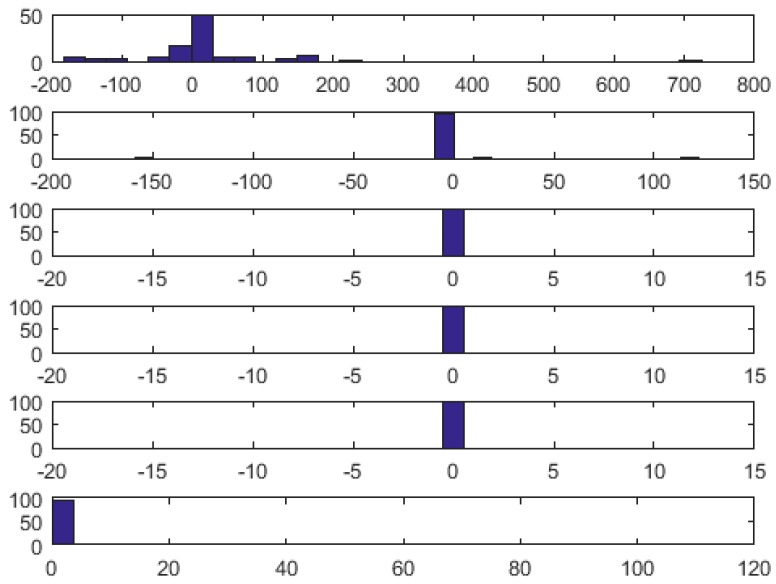
Histograms of QRS length error distribution for db5 wavelet and *n* = {1…5} bit depth watermark coding (top to bottom); the lowest plot shows the automatic adjustment of coding bit depth.

Table 3 reveals that watermark coding bit depth equal to 4 (corresponding to noise amplitude of 4 µV) is the most appropriate. Independently on the wavelet applied, in all cases, it produces transparent (i.e., error free, preserving all medical features) stegano ECG. In case of bior4.4 and sym11 wavelets with coding bit depth equal to 3, and in the case of db5 and bior2.4 wavelets with coding bit depth equal to 5 the watermarking also produced transparent stegano ECG. It is noteworthy that most significant errors, including all cases exceeding IEC accuracy requirements, occur for 1-bit coding. In this case, the time-frequency representation of the ECG is simplified too much, and some energy of ECG waves is also truncated. Similar conclusions can also be drawn from histograms of intervals length error distribution, such as the one presented in Figure 7.

With automatic adjustment of coding bit depth, the watermarking performed slightly worse than with the fixed value of 4, however the difference was hardly noticeable. The efficiency of the adjustment process requires test ECGs with controlled noise level and, thus, was not tested in our experiment because of the use of CSE MA files with all identical heartbeats.

### 5.4. Results for Different Watermark Content 

The values of the watermark are first chopped into given *n*-bit resolution samples which replace the noise values in the data container part of original time-scale ECG representation. Therefore, statistical distribution of the watermark values influences the transparency of the watermarking process. This is visible in Table 1 as different mean error values for textual (average 1.22 ms, error-free 78 cases out of the total 144) and numerical (average: 1.35 ms, error-free: 71 cases) watermarks, and motivated us to make pairwise comparisons of 100-elements error sets between two tested watermark contents using a *t*-test. 144 such comparisons were done iterating through four interval types, six wavelet types and six values of coding bit depth (including the automatic one). The example result for QRS complex length is presented in Table 4.

Apart from 4-bit coding producing error-free stegano ECG for all tested wavelets, improper setting of coding bit depth results in differences between textual and numerical watermarks in most cases (significant non-zero *t* values). Short wavelets: db5, sym6 and bior2.4 seem to be more tolerant to the changes of watermark values distribution than longer wavelets.

### 5.5. Results for Different ECG Content 

The IEC-recommended subset of CSE-files contains 100 ECG records of atrial rhythm (with present P wave) of different heart rates. The heart rate, however, does not proportionally shorten or lengthen individual sections of the heartbeat. Therefore, it is worth analyzing the influence of the heart rate to the watermark capacity and the average ECG distortion expressed accordingly to IEC as interval measurement error. The result of such analysis is presented in Table 5.

Apart from the heart rate dependence, several files in the testing set were systematically found as the source of errors. Six such files with their brief characteristics are described in Table 6. 

## 6. Discussion

The paper presents an original concept of wavelet-based irreversible watermarking of ECG based on expected variations of local bandwidth related to P, QRS and T wave positions. The novelty of our method also included continuous noise measurement and adaptation of the coding bit depth in order to best imitate the intrinsic ECG noise. The watermarking technique was implemented and was then thoroughly tested according to industrial standards of performance that are required for automated interpretive software. Consequently, the safe use area was determined where the watermarking does not change the medical content of the host ECG. We proved that the selection of the wavelet and the adjustment of the coding bit depth both play important roles in the preservation of watermarking transparency. Moreover, we found that the distribution of watermark values influences the possible error rate and that some ECG files are less suitable for hosting a watermark due to their content.

For the evaluation of the watermarking, we consequently desisted from using the PRD and other purely statistical signal metrics, as loosely related to the medical content of ECG. Average distortion level that is measured by additional error of interval length measurement (Table 5) is acceptable (below 1 ms for P and P-Q, 1.2 ms for Q-T and 2.05 ms for QRS). The last value is comparable to a single sampling interval of the ECG and results mainly from inaccuracy of QRS-end calculation. Other wave border points, in particular P-onset, P-end and QRS-onset are far less influenced by watermarking.

Although the PRD is commonly questioned as a comparative ECG metrics due to disregarding the distribution of medical content, we calculated its value for a single reason of juxtaposition of the watermarking transparency with other known methods (Table 7). In this paper, we already proved the dependence of the watermarking performance on the wavelet used, coding bit depth, watermark and ECG content. A fair comparison of literature methods should additionally take into consideration various watermark capacity, coding conditions and host ECG data sets that were used by the authors.

The use of a specific interpretive software in the assessment of the proposed watermarking method is probably the most controversial issue of this paper. We assume that for each wave border point, the position measurement error consists of two independent values: one is caused by the interpreting software, while the other is due to the watermarking process. The CSE database provides measurement results from real software packages and experts with an assumption that their mean value is closer to the truth (which remains unknown) than any single result. Thanks to decades of engineering effort, some recent packages shows the measurement precision below 1 ms in manufacturer-independent tests. Unfortunately, even the best (and most expensive) software is not a reference of absolute accuracy. Moreover, ECG recorders in which it would be built-in, rarely accept external records in well documented SCP-ECG format for interpretation. For this reason, we have taken IEC certified package from Ascard 6, available to us courtesy of Aspel S.A., and considered results that were obtained for watermark-free records (i.e., CSE MA files) as the reference, not regarding how far they are from the CSE mean values. Consequently, all measurement errors we report in this paper are differences between results from watermarked records and from non-watermarked files that were returned by the same interpretive procedures. Despite our best care of repeatability of research, we are conscious that using other interpretive software may yield slightly different results.

The Symlet of 11-th order (sym11) seems to be the most adequate for watermarking of all the wavelet types we tested. The reason for this is its similarity to the ECG component (best of all ‘long’ wavelets) and good separation of energy between scales (best of all ‘short’ wavelets). Thanks to the similarity of the temporal waveform, the ECG is well represented in few neighboring time-frequency coefficients without too much of energy spread in time, which also yields low interference from the watermark to the high frequency components of P and QRS waves. At the same time, the sym11, being a relatively long filter, has steeper frequency slopes and separates the low frequency content of T wave (4th scale and below) from the influence of the watermark container (1st scale) and its description (2nd scale). The sym11 complies with IEC accuracy requirement in a wide range of coding bit depth and watermark and host ECG content. However, it cannot be excluded that:

- further research will show another wavelet performing even better,
- specific ECG content (e.g., variety of ventricular beat shapes) or different ECG sampling rate will need other wavelet for optimal representation.

Preparing of watermark content plays a crucial role in maintaining the transparency of the process. Coding the watermark with inappropriate bit depth results in serious deterioration of the ECG content. For this purpose, we used a simple noise level tracking procedure, which seems to be sufficient in stable noise conditions. In the future, we aim to improve the noise analytics and implement a dynamic regularization procedure to make the watermark content even more like the intrinsic noise.

Most of the literature reports suggest using the watermark for integration and storage of patient demographic or medication data. Following this hint, we tested our original method with textual and numerical data strings. Nevertheless, watermarks can also be created of data of various types, therefore, including binary data of any specific distribution is planned in future tests. Furthermore, a watermark regularization procedure is expected to alleviate the problem of watermark value distribution. 

The tested irreversible watermarking method proved to provide a stable watermark capacity of ca. 140 samples per second (Table 5). Assuming stable ECG noise level of order of 4 µV and, consequently, watermark coding at 4-bit per sample, this means a 560 bit per second (bps) data rate in a single lead (i.e., 6.56 kbps in a 12-lead record), which seems to be sufficient to accommodate not only administrative data, but also the results of accompanying measurements (from the environment or from the patient himself). However, further processing of the stegano ECG subjects to some limitations:

- bit-accurate (lossless) compression preserves the watermark, however it may be less effective,
- lossy compression methods destroy the watermark, unless purposely designed,
- further watermarking destroys the existing watermark (watermarking with meaningless data may be used to safely remove the existing watermark),
- inter channel dependencies (e.g., the triangle relation allowing for the calculation of any limb lead from two others) are lost.

It is noteworthy that watermark containers are independent data structures, however they may be linked to increase the capacity. Data within each container or linked containers have to be uniform. Otherwise, different data structures may carry various data types and, with appropriate cryptographic techniques, may require a reader-specific authorization.

Making tests with the IEC-recommended subset of CSE files, we prove compliance of the watermarking process to the industrial performance standard. In fact, the IEC 60601-2-51 document requires testing wave delimitation accuracy only with atrial rhythms, where all P, QRS and T waves are present. Besides the normal ECG, the testing set includes both Bundle Branch Blocks, Hypertrophy, Wolf-Parkinson-White Syndrome and many others (see [30,31] for the list of the represented medical cases). In the case of Ventricular beats, included in the CSE Database, however not in the testing set, the watermarking algorithms work fine as long as wave delineation is reliable. The same is observed for arrhythmia patterns like bigeminy or ventricular pairs.

However, we found some limitations of the method, even in records of regular atrial beats (see Table 6). Moreover, in the case of Atrial Fibrillation or Flutter (continuous P-like wave), Ventricular Fibrillation (no distinct QRS activity) or Acute Infarct with ST Elevation (STEMI) where no consistent QRS-end can be determined, as well as in the case of pacemaker records, the proposed watermarking method cannot be performed.

The algorithm may also be working inappropriately in the case of Ventricular Late Potentials (high frequency components at the end of the QRS) and, in the presence of T-wave alternans, watermarks may interfere the T-wave contents, making identification of alternans patterns less reliable. These two cases, however, need further studying. Future variants of ECG analysis preceding the actual watermarking needs to be extended towards automatic detection and exclusion of ECG patterns where the watermark embedment is not advisable.

## Figures and Tables

**Table 1:** Average difference values [ms] between results on IEC interval length calculated from 100 clean and watermarked ECGs; gray background indicates cases with identical results; boldface indicates differences exceeding the IEC requirement (10 ms for P, P-Q, QRS and 30 ms for Q-T); letter ‘A’ in coding bit depth column stands for automatic adjustment dependent on noise level.

Wavelet	Bit Depth	Numerical Watermark				Textual Watermark			
		P	PQ	QRS	QT	P	PQ	QRS	QT
sym6	1	6.41	7.28	**12.94**	1.16	**10.59**	**10.40**	**12.54**	−4.91
	2	−1.29	−1.65	0.19	0.20	−1.29	−1.65	0.60	0.01
	3	0.00	0.00	0.00	0.00	−0.79	−1.22	0.18	0.25
	4	0.00	0.00	0.00	0.00	0.00	0.00	0.00	0.00
	5	0.00	0.00	0.05	−0.09	0.00	0.00	0.05	−0.09
	A	0.00	0.00	0.05	−0.09	0.00	0.00	0.05	−0.09
sym11	1	0.72	0.45	4.87	−4.96	−3.41	−3.76	2.31	−4.40
	2	−1.54	−1.87	0.98	0.20	-0.30	-0.60	−0.12	−0.27
	3	0.00	0.00	0.00	0.00	0.00	0.00	0.00	0.00
	4	0.00	0.00	0.00	0.00	0.00	0.00	0.00	0.00
	5	−0.14	−0.09	0.10	0.04	0.00	0.00	0.00	0.00
	A	0.00	0.00	0.00	0.00	0.00	0.00	0.01	0.00
bior2.4	1	2.89	2.18	**19.70**	−7.22	−3.19	−3.27	**16.40**	−6.65
	2	−2.08	−2.87	−1.21	−1.92	−1.29	−1.65	0.37	0.20
	3	−0.79	−1.22	0.18	0.25	−0.79	−1.22	0.18	0.25
	4	0.00	0.00	0.00	0.00	0.00	0.00	0.00	0.00
	5	0.00	0.00	0.00	0.00	0.00	0.00	0.00	0.00
	A	0.00	0.00	0.00	0.00	0.00	0.00	0.00	0.00
bior4.4	1	−5.56	−3.73	**16.74**	−9.74	−1.57	−0.97	**16.50**	−2.48
	2	−1.36	−1.97	−1.08	−3.25	−1.36	−1.97	0.55	−0.97
	3	0.00	0.00	0.00	0.00	0.00	0.00	0.00	0.00
	4	0.00	0.00	0.00	0.00	0.00	0.00	0.00	0.00
	5	0.00	0.00	0.05	−0.09	0.00	0.00	0.00	0.00
	A	0.00	0.00	0.05	−0.09	0.00	0.00	0.00	0.00
db5	1	−0.03	−0.30	**16.68**	−5.87	−3.34	−3.56	**14.16**	−5.09
	2	−2.08	−2.87	0.49	0.63	3.53	3.12	−0.86	−1.82
	3	−0.79	−1.22	0.18	0.25	0.00	0.00	0.00	0.00
	4	0.00	0.00	0.00	0.00	0.00	0.00	0.00	0.00
	5	0.00	0.00	0.00	0.00	0.00	0.00	0.00	0.00
	A	0.00	0.00	0.00	0.00	0.00	0.00	0.00	0.00
db10	1	1.01	−0.30	7.26	−15.27	−0.46	−1.89	5.56	−7.73
	2	−0.59	−0.68	0.01	−0.10	−0.59	−0.68	−0.08	−0.99
	3	−1.24	−1.27	0.83	0.46	0.00	0.00	0.00	0.00
	4	0.00	0.00	0.00	0.00	0.00	0.00	0.00	0.00
	5	0.00	0.00	0.00	0.00	−0.14	−0.09	0.10	0.04
	A	0.00	0.00	0.01	0.00	0.00	0.00	0.01	0.00

**Table 2:** Watermarking performance with use of different wavelets (40 iterations for 4 intervals, coding bit depth from 1 to 5 and textual or numerical watermark).

Wavelet Type	Sum of Average Errors (ms)	Number of Errorless Cases	Errors Exceeding IEC Requirements
sym11	31.13	20	0
db10	47.37	16	0
db5	66.87	20	2
bior4.4	69.94	22	2
sym6	75.83	16	4
bior2.4	77.97	16	2

**Table 3:** Watermarking performance with use of different watermark coding bit depth value (48 iterations for 4 intervals, six wavelets types and textual or numerical watermark).

Coding Bit Depth (Bits per Sample)	Sum of Average Errors (ms)	Number of Errorless Cases	Errors Exceeding IEC Requirements
1	298.41	0	10
2	55.98	0	0
3	13.56	28	0
4	0	48	0
5	1.16	34	0
automatic	0.45	39	0

**Table 4:** *T*-test value (confidence level *p* = 0.05) of pairwise comparisons of 100-elements error sets between numerical and textual watermark contents; the interval measured was QRS complex.

Wavelet	Coding Bit Depth					
	1	2	3	4	5	Auto
db5	1	1	0.8413	0	0	0
db10	1	1	1	0	0.1587	0.5
sym6	1	0.1585	0.1587	0	0.5	0
sym11	1	1	0	0	0.8413	0.1587
bior2.4	1	0	0.5	0	0	0
bior4.4	1	0	0	0	0.8413	0.8413

**Table 5:** Influence of the host ECG heart rate to the watermark capacity and the average interval measurement error.

RR Value Range (ms)	No. of IEC Files	Watermark Container Capacity (Samples)		Average Interval Measurement Error (ms)			
		per Container	per Second	P	PQ	QRS	QT
<600	8	80	140.5	0.97	0.91	1.77	1.26
600–700	21	95	139.8	0.91	1.00	1.96	1.04
700–800	32	113	143.1	0.79	0.83	2.32	1.44
800–900	18	127	141.7	0.77	0.78	2.12	1.18
900–1000	14	138	142.9	0.86	0.94	2.38	1.37
>1000	7	144	142.3	0.83	0.89	1.89	1.08

**Table 6:** Analysis of CSE files causing outlying results in the test

Error Type	CSE File No.	Characteristics
important number of significant errors (exceeding IEC tolerance)	12	significant level of high frequency noise; when discriminated in low bit depth, watermarking leads to differences in wave borders calculation
	13	low amplitude of P and T waves; positioning of wave borders is tentative and alters significantly in low bit depth watermarking
important number of minor errors (within IEC tolerance)	7	significant noise level at the baseline; systematic wave positioning error occurs for 1- and 2-bit depth coding
	61	rhythmic sharp artifacts lead to unstable positions of wave borders; watermarking causes random discrimination of some of these components and alternates the wave positioning result
occasional significant errors (exceeding IEC tolerance)	14	smooth QRS ending slope causes errors in QRS-end detection and results in watermark container occasionally overlaps QRS components
	81	important power line artifact not following the expected noise spectrum; for 1-bit watermarking with db5, sym6 and bior4.4, the stegano ECG becomes useless

**Table 7:** Juxtaposition of the watermarking transparency expressed by PRD value for selected literature methods and this work

Work	PRD (%)	Comment
Wang [8]	0.000	reversible watermarking, 20 arrhythmia ECG files from Massachussetts Institute of Technology-Beth Israel Hospital (MIT-BIH) Database [36]
Jero [22]	0.006–0.036	561 bytes patient data is embedded into a 76,800 samples ECG strip, MIT-BIH Database files 101–109
Jero [23]	0.007–0.11	83–502 bytes patient data is embedded into a 76,800 samples ECG strip, unspecified MIT-BIH Database file
Ibaida [17]	0.02–0.32	bit depth 2–5; 81 normal ECG segments from The Creighton University Ventricular Tachyarrhythmia Database [36]
this work	0.06–0.51	bit depth 2–5; 100 atrial ECG files from CSE Multilead Dataset 3
Awarsamol [27]	0.22–0.64	measured in time-scale domain, three unspecified Physionet records
Ibaida [18]	0.35–0.81	18 normal sinus rhythm 10-s ECG files from custom-recorded database
